# A Case of Complete Functional Myocardial Regeneration in a Human Neonate

**DOI:** 10.1016/j.jaccas.2025.103622

**Published:** 2025-06-11

**Authors:** Jyoti Gur, Rajiv Devanagondi, Rebecca Pratt, Frank Smith, Jason Mandell, Susan Martin, George A. Porter

**Affiliations:** a Department of Pediatrics, Division of Pediatric Cardiology, University of Rochester, Rochester, New York, USA; b Department of Pediatrics, Division of Pediatric Cardiology, University of Buffalo, Buffalo, New York, USA; c Division of Pediatric Cardiology, SUNY Upstate Medical University, Syracuse, New York, USA; d Department of Pediatrics, Division of Critical Care, University of Rochester, Rochester, New York, USA

**Keywords:** cardiac regeneration, congenital heart disease, hypoxemia, ischemia

## Abstract

**Background:**

Multiple studies demonstrate the cardiac regenerative capacity in neonatal mice in the first week of life. Although a similar regenerative window in the human neonatal hearts has not been defined clearly, existing evidence supports the concept of human neonatal cardiac regeneration.

**Case Summary:**

A neonate with a complex form of cyanotic tetralogy of Fallot demonstrated complete recovery of left ventricular function after an acute cardiac injury suffered on day of life 8.

**Discussion:**

This case report adds to limited but intriguing observations that human neonatal hearts may possess the regenerative capacity observed in animal models and that that capacity may be prolonged by hypoxemia.

**Take-Home Messages:**

A human neonatal heart has shown complete functional recovery after an ischemic injury. Hypoxemia may have extended the regenerative window of the human neonatal myocardium.

## Visual Summary

**Figure:**
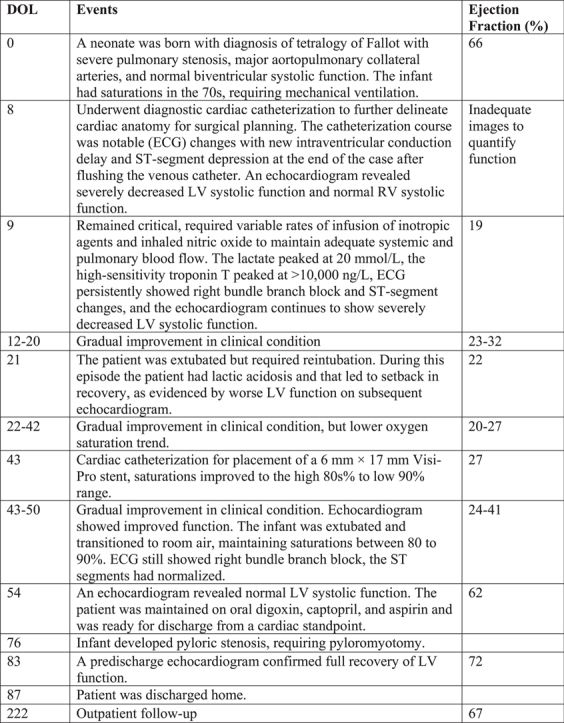

## History of Presentation

**Figure 1:**
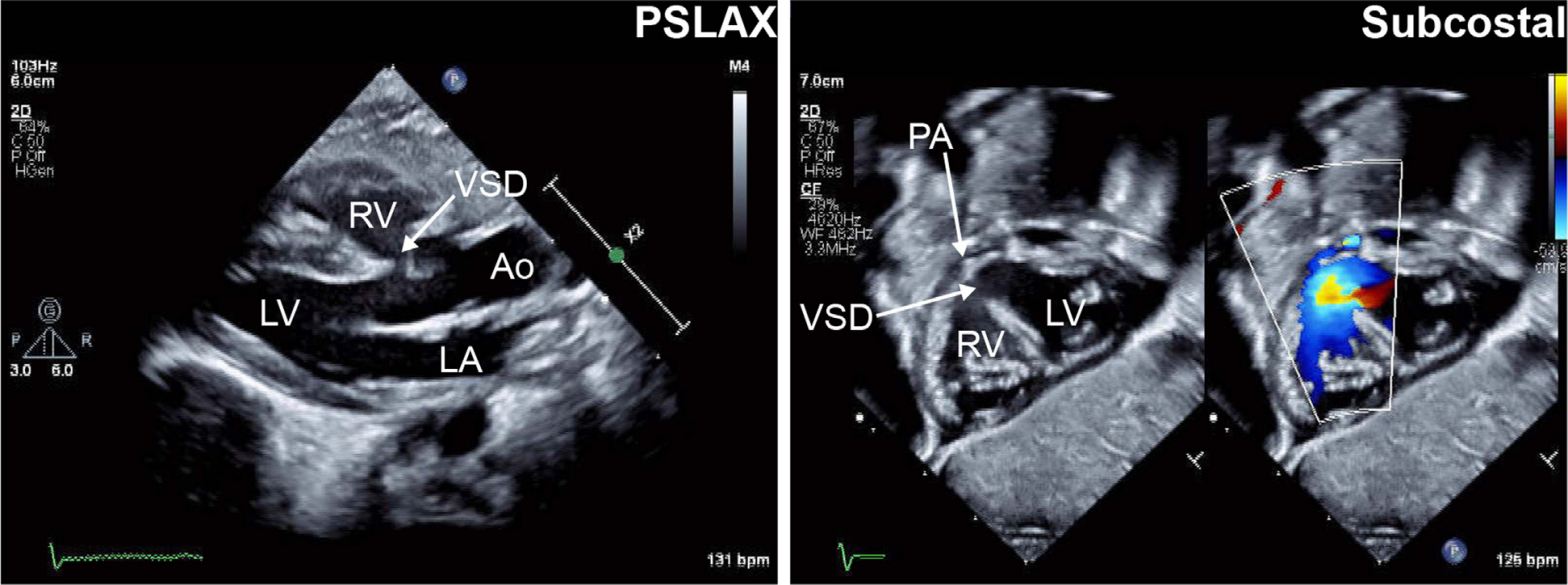
Echocardiograms Demonstrating Tetralogy of Fallot With Severe Pulmonary Stenosis Parasternal long axis and subcostal sagittal images demonstrate the patient’s anatomy. Color flow imaging in the subcostal image shows severe subpulmonary and pulmonary stenosis and right to LV level shunt. Depth (cm) is indicated to the right of each image. Ao = ascending aorta; LA = left atrium; LV = left ventricle; PA = pulmonary artery; RA = right atrium; RV = right ventricle; VSD = ventricular septal defect.

A 17-year-old mother who received adequate antenatal care and had no prenatal complications and normal prenatal laboratory test results and sonograms gave birth by spontaneous vaginal delivery to a 2.8 kg girl at 39 weeks of gestation. The infant had meconium-stained amniotic fluid and was noted to have oxygen saturations in the 70s immediately after birth despite receiving 100% oxygen and normal work of breathing. The patient was intubated and mechanically ventilated. An echocardiogram revealed tetralogy of Fallot with severe pulmonary stenosis with a bicuspid and thickened pulmonary valve, severely hypoplastic and confluent main and branch pulmonary arteries with retrograde filling of the left pulmonary artery, major aortopulmonary collateral arteries, no definite ductus arteriosus, normal biventricular systolic function, and a right aortic arch with mirror image branching (Figure 1). The cyanosis was thought to be due to limited pulmonary blood flow and not lung disease, and the infant was initiated on infusions of epinephrine and dopamine and norepinephrine (0.05, 5-7.5, an 0.04 μg/kg/min, respectively) for 1 to 2 days to support pulmonary blood flow and a prostaglandin E1 infusion (dose 0.05 μg/kg/min), which seemed to increase oxygen saturation slightly.

**Take-Home Messages**

• A neonatal with cyanotic heart disease had complete functional recovery of the left ventricle after left coronary artery thrombosis at 8 days of age.
• The temporal window for cardiac regeneration in the human neonatal heart may be extended by hypoxemia.
• This case study provides new insights into the field of neonatal cardiac biology and myocardial regeneration and suggests further similarities between mouse models of this process and human disease.

**Figure 2:**
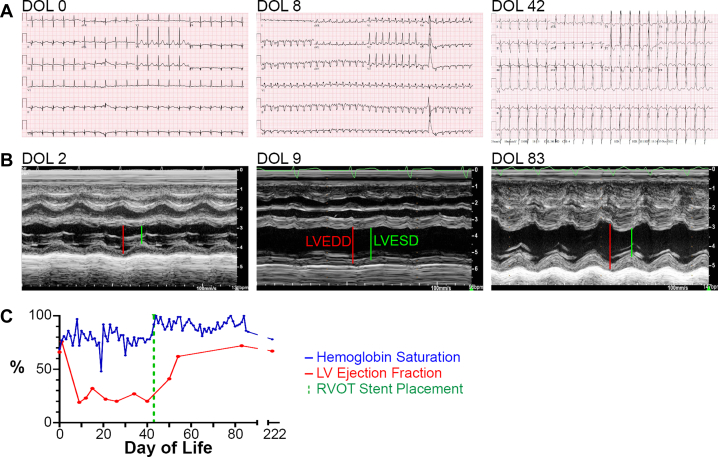
Effects of Cardiac Injury on ECG Morphology and Cardiac Function (A) ECGs at different days of life demonstrate normal ST-segment on DOL0, tachycardia, new intraventricular conduction delay, and diffuse ST-segment changes on DOL8, and normalization of ST segment changes with persistent conduction delay on DOL42. (B) Parasternal short axis M-mode images demonstrate normal LV function on DOL2, severely depressed LV systolic function on DOL9, and return to normal LV function on DOL83. Lines represent LV end-diastolic (LVEDD, red) and end systolic (LVESD, green) dimensions, and depth [cm] is indicated to the right of each image. (C) Graphical representation of hemoglobin saturation by pulse oximetry (blue line) and LV ejection fraction (red line, measured by standard 5/6 area-length technique in the apical 4-chamber view) during the patient’s course demonstrates acute, severe dysfunction at DOL8 with recovery to normal starting at DOL32. The RVOT stent was placed on DOL43 (green dashed vertical line). LV = left ventricle; RVOT = right ventricular outflow tract.

Over the next 8 days, the neonate was stabilized and on day of life (DOL) 8 (weight 3.29 kg) underwent diagnostic cardiac catheterization to further delineate cardiac anatomy for surgical planning. The study revealed a severely hypoplastic pulmonary valve without antegrade flow, severely hypoplastic but confluent branch pulmonary arteries, and multiple very small aortopulmonary collaterals from the subclavian arteries, thyrocervical trunk, and descending thoracic aorta. The study revealed no patent ductus arteriosus, so PGE was discontinued. A catheter could not be advanced passed the pulmonary valve precluding right ventricular outflow tract (RVOT) stenting. The catheterization course was notable for acute electrocardiogram (ECG) changes with new intraventricular conduction delay and ST-segment depression late in the case after flushing the venous catheter (Figure 2A). Upon return to the intensive care unit, the patient was hemodynamically unstable, and an echocardiogram revealed severely decreased left ventricular (LV) systolic function and normal RV systolic function (Figures 2B and 2C, Video 1). This LV dysfunction was presumed to be secondary to a thromboembolic event leading to left coronary artery insufficiency, although the proximal origins of the coronary arteries were visualized in the echocardiogram and seemed to be patent. Over the next day, the patient required variable infusion rates of inotropic agents (epinephrine up to 0.1 μg/kg/min, dopamine up to 10 μg/kg/min, and milrinone at 0.25 μg/kg/min) and inhaled nitric oxide to balance systemic and pulmonary blood flow. Hemodynamic instability and body weight precluded a return to the catheterization laboratory for coronary angiography and local thrombolytic therapy, but a heparin infusion was initiated. Over the next 24 hours, the lactate peaked at 20 mmol/L, the high-sensitivity troponin T peaked at >10,000 ng/L (Table 1), and the ECG persistently showed right bundle branch block and ST-segment changes (Figure 2A). Dopamine and epinephrine were weaned off after 3 and 5 days, respectively. Owing to the anatomy and hospital course, the infant was deemed not a candidate for pulmonary artery unifocalization, shunt placement, or extracorporeal membrane oxygenation, and the parents requested no chest compressions, cardioversion, or defibrillation. The patient remained on a therapeutic heparin infusion for next 4 days.

**Table 1:** Cardiac Biomarkers Indicate Acute Cardiac Injury and Systemic Hypoperfusion

	DOL8 (3 h After Injury)^a^	DOL8 (6 h After Injury)	DOL9 (24 h After Injury)	DOL10 (48 h After Injury)	DOL54
hsTnT (0-14 ng/L)	852	6,399	>10,000		69
Lactate (0.3-0.8 mmol/L)	3.3	19.7	13.4	2.5	0.9

DOL = day of life; hsTnT = high-sensitivity troponin T, reference ranges in parentheses.

a Time represented in DOL and hours after injury on DOL8.

Over the next 2 weeks, the patient showed continuous improvement. Lactate levels trended down to the range of 1 to 2 mmol/L (Table 1), and oxygenation remained stable within goal range of 70% to 80% on minimal ventilatory support with 30% to 50% F_i_O_2_ (Figure 2C). The patient was weaned off all inotropic agents except milrinone gradually. Serial echocardiograms during this period showed slight recovery in LV function (ejection fraction [EF] of 32% on DOL15) (Figure 2C). The infant maintained normal renal function and adequate urine output. Feeding through a nasogastric tube was well tolerated. Magnetic resonance imaging of the brain performed 10 days after the initial event revealed acute to early subacute infarcts in the right basal ganglia and temporal occipital lobe, resulting from a thromboembolic event, with no evidence of hemorrhagic transformation, midline shift, or mass effect.

Encouraged by overall improvement, the patient was extubated on DOL21 but required reintubation owing to poor cardiac output and lactic acidosis, and LV function was worse on a subsequent echocardiogram (DOL26, EF of 20%) (Figure 2C). In this period, the patient exhibited a downtrend in saturations and required increased oxygen support, discontinuation of milrinone and initiation of norepinephrine and vasopressin (0.12 and 0.06 μg/kg/min, respectively) for 7 days. Echocardiograms demonstrated a qualitative increase in the volume of antegrade pulmonary flow, together with a decrease in collateral flow. Thus, the patient underwent pulmonary balloon valvuloplasty and RVOT stenting (6 × 17 mm) on DOL43, which immediately increased the saturations to the low 90% range. On DOL50, an echocardiogram showed improvement of LV dysfunction to the mild-to-moderate range, so milrinone was discontinued (Figure 2C). The infant was extubated and transitioned to room air, maintaining saturations between 80% and 90%. Although the ECG still showed right bundle branch block, the ST-segment had normalized (Figure 2A).

On DOL54, an echocardiogram revealed normal LV systolic function, indicating complete recovery of previously depressed LV function. The patient was maintained on oral digoxin, captopril, and aspirin and was ready for discharge from a cardiac standpoint, but developed pyloric stenosis requiring pyloromyotomy on DOL76. After recovering from the surgery, the patient was discharged home on DOL87, breathing room air with oxygen saturations in the 80% to 90% range. A pre-discharge echocardiogram confirmed full recovery of LV function (Figures 2B and 2C, Video 2).

## Discussion

The infant described demonstrated complete recovery of LV function after sustaining a life-threatening cardiac injury on DOL8. Although we present only indirect evidence of ischemic myocardial injury in this patient, the presence of acute diffuse ST-segment changes on the ECG (Figure 2A), elevation of cardiac biomarkers (Table 1), severe deterioration in LV function with preservation of RV function (Figure 2B), and multiple infarcts on magnetic resonance imaging of the brain suggest a thromboembolic event in the left coronary artery. The patient likely survived because the RV was able to sustain systemic and pulmonary output owing to the ventricular septal defect. This case was further complicated by hypoxemia from tetralogy of Fallot, near pulmonary atresia, and major aortopulmonary collateral arteries. However, pulmonary blood flow was later stabilized by placing a stent in the RVOT. In the presence of global LV hypokinesis and paradoxical ventricular septal motion, exact quantification of the of LV EF by two-dimensional echocardiography was difficult. For this reason, we did not have EF values for every echocardiogram, but the patient showed some recovery in LV function (EF of 32%) on DOL15. There was a setback in this early recovery period owing to failed extubation, which compromised systemic perfusion. However, the LVEF remained decreased until after the stent was placed on DOL43 (Figure 2C). It is possible that this recovery was related to stent placement and the subsequent increase in saturation. Placement of the stent could also have improved hemodynamic interventricular interaction, but throughout the entire period we report on, the function of the RV remained qualitatively normal and not affected by the patient's state of hypoxemia or the stenting of its outflow tract. Furthermore, many infants with a similar degree of cyanosis owing to a heart defect do not exhibit poor ventricular function, while the timing of recovery can be explained by coronary recanalization and/or collateralization followed by regeneration. Therefore, it is entirely possible that regeneration explains the recovery of LV function.

The concept of human cardiac regeneration has always interested researchers. In 1937, McMahon found mitotic nuclei within cardiomyocytes from 4 children ranging in age from 6 months to 6 years. Based on these findings and a review of literature from the previous century, McMahon postulated that young mammalian hearts could regenerate.^1^ Although patients with anomalous left coronary artery from the pulmonary artery have shown functional recovery of LV function after surgical reestablishment of dual coronary circulation, the extent of recovery likely depends on the degree of coronary collateralization and age of repair and may not depend on true regeneration.^2^ Haubner et al^3^ recently published a report of functional recovery of a human neonatal heart after severe myocardial infarction immediately after birth and cited a few similar reports from the previous 3 decades. Experiments in neonatal mice and other animal models have shown a brief window of regenerative capacity in the early neonatal period.^4^^,^^5^ This period of myocyte proliferation and/or regeneration can be extended by various factors, such as hypoxemia, antioxidants, inhibition of the DNA damage response, neuregulin, adrenergic inhibition, and manipulation of other pathways or conditions.^6^, ^7^, ^8^, ^9^ Studies have also shown that neuregulin increased proliferation in myocardium cultured from humans up to 6 months of age, whereas propranolol increased proliferation of myocytes cultured from patients with tetralogy of Fallot.^10^^,^^11^ Therefore, perhaps beta-adrenergic blockade may have been more efficacious to treat the patient’s heart failure, and this is the subject of a current clinical trial (NCT04713657).^11^

## Conclusions

This patient’s recovery adds to the few reports of neonatal regeneration in humans and further supports the idea that hypoxemia may prolong this period of human cardiac regeneration, as it does in mice.

**Visual Summary:** Timeline of Case

DOL	Events	Ejection Fraction (%)
0	A neonate was born with diagnosis of tetralogy of Fallot with severe pulmonary stenosis, major aortopulmonary collateral arteries, and normal biventricular systolic function. The infant had saturations in the 70s, requiring mechanical ventilation.	66
8	Underwent diagnostic cardiac catheterization to further delineate cardiac anatomy for surgical planning. The catheterization course was notable for (ECG) changes with new intraventricular conduction delay and ST-segment depression at the end of the case after flushing the venous catheter. An echocardiogram revealed severely decreased LV systolic function and normal RV systolic function.	Inadequate images to quantify function
9	Remained critical, required variable rates of infusion of inotropic agents and inhaled nitric oxide to maintain adequate systemic and pulmonary blood flow. The lactate peaked at 20 mmol/L, the high-sensitivity troponin T peaked at >10,000 ng/L, ECG persistently showed right bundle branch block and ST-segment changes, and the echocardiogram continues to show severely decreased LV systolic function.	19
12-20	Gradual improvement in clinical condition	23-32
21	The patient was extubated but required reintubation. During this episode the patient had lactic acidosis and that led to setback in recovery, as evidenced by worse LV function on subsequent echocardiogram.	22
22-42	Gradual improvement in clinical condition, but lower oxygen saturation trend.	20-27
43	Cardiac catheterization for placement of a 6 mm × 17 mm Visi-Pro stent, saturations improved to the high 80s% to low 90% range.	27
43-50	Gradual improvement in clinical condition. Echocardiogram showed improved function. The infant was extubated and transitioned to room air, maintaining saturations between 80 to 90%. ECG still showed right bundle branch block, the ST segments had normalized.	24-41
54	An echocardiogram revealed normal LV systolic function. The patient was maintained on oral digoxin, captopril, and aspirin and was ready for discharge from a cardiac standpoint.	62
76	Infant developed pyloric stenosis, requiring pyloromyotomy.	
83	A predischarge echocardiogram confirmed full recovery of LV function.	72
87	Patient was discharged home.	
222	Outpatient follow-up	67

DOL = day of life; ECG = electrocardiogram; LV= left ventricle; RV = right ventricle.

## Funding Support and Author Disclosures

The authors have reported that they have no relationships relevant to the contents of this paper to disclose.

## Appendix

Video 1 Echocardiogram Severely decreased LV function on DOL8. Two beat clip of parasternal short axis view shows severely decreased LV function (lower chamber) and normal RV function (upper chamber) on DOL 8 after the ischemic injury.

Video 2 Echocardiogram Normal LV function on DOL83. Three-beat clip of parasternal short axis view shows normal LV (lower chamber) and RV (upper chamber) function on DOL83.
